# Correction: Apolipoprotein E intersects with amyloid-β within neurons

**DOI:** 10.26508/lsa.202402875

**Published:** 2024-06-21

**Authors:** Sabine C Konings, Emma Nyberg, Isak Martinsson, Laura Torres-Garcia, Oxana Klementieva, Claudia Guimas Almeida, Gunnar K Gouras

**Affiliations:** 1 Experimental Dementia Research Unit, Department of Experimental Medical Science, Lund University , Lund, Sweden; 2 Medical Microspectroscopy, Department of Experimental Medical Science, Lund University , Lund, Sweden; 3 iNOVA4Health, NOVA Medical School | Faculdade de Ciências Médicas, Universidade Nova de Lisboa, Lisboa, Portugal

## Abstract

Apolipoprotein E4, the most important genetic risk factor for Alzheimer's disease, is shown to internalize into neurons and intersect with amyloid-β in endosomes-autophagosomes of neurites and modulate intraneuronal amyloid-β-42.

Article: Konings SC, Nyberg E, Martinsson I, Torres-Garcia L, Klementieva O, Guimas Almeida C, Gouras GK (2023 Jun 8) Apolipoprotein E intersects with amyloid-β within neurons. Life Sci Alliance 6(8): e202201887. doi: 10.26508/lsa.202201887. PMID: 37290814.

Following publication, the authors noticed that a subfigure in Fig S3 contained incorrect images; the incorrect images have now been replaced by the correct images in the included new Fig S3. The Supplemental figure that is being corrected relates to the main Fig 2, which includes images for 1 h of Bafilomycin A1 treatment followed by 4 h of ApoE treatments, while two subfigures in Fig S3 just contained extended time points of 8 and 24 h of ApoE treatments to underscore that even long incubations gave similar results. Specifically, subfigure Fig S3E had three examples of 8 h of ApoE treatments, which are all correct, while the faulty Fig S3K with the 24 h time-points of ApoE treatments had incorrect images that are now replaced with the correct ones in this corrected Fig S3. There is no change needed for the manuscript text or to the figure legends. This error does not affect the conclusions of this manuscript. The incorrect images were inadvertently used in the resubmission of this manuscript.

**Figure:**
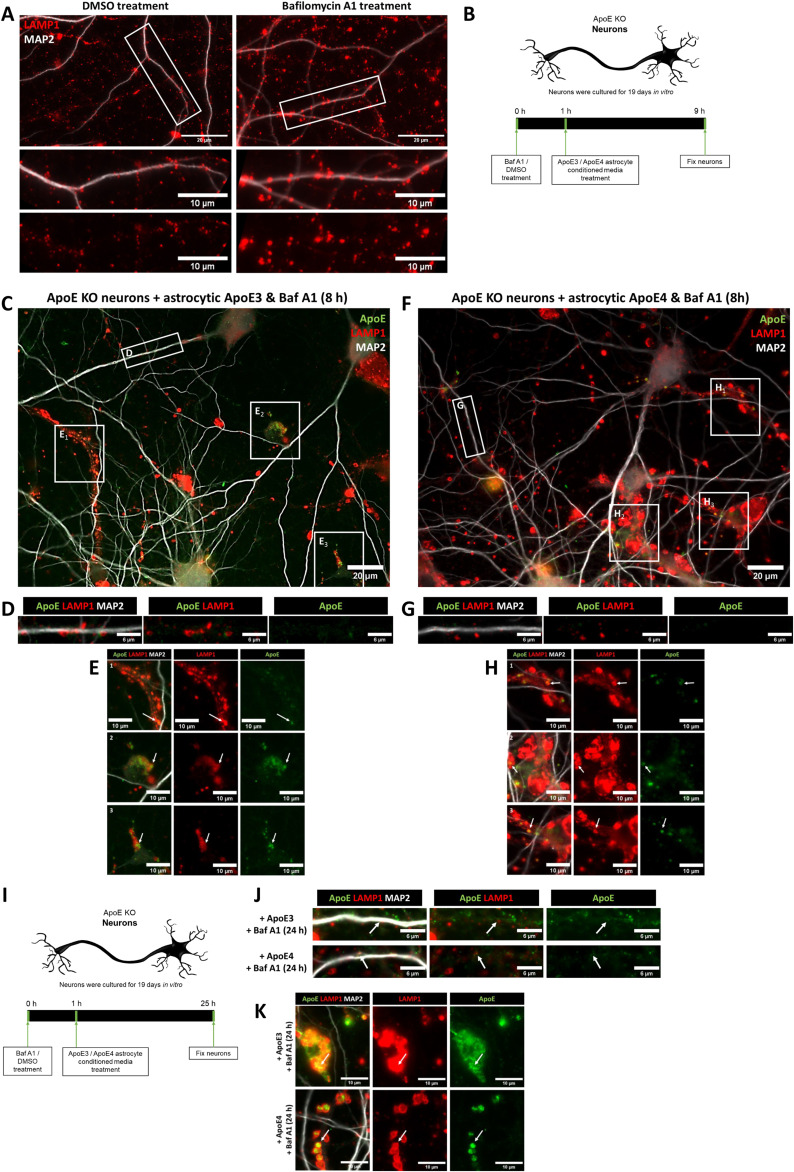


## Supplementary Material

Reviewer comments

