# Supplementary material for: Correction: Apolipoprotein E intersects with amyloid-β within neurons
Source: Life Sci Alliance. 2024 Jun 21;7(9):e202402875. doi: 10.26508/lsa.202402875 (PMC11192963; doi:10.26508/lsa.202402875)
Supplement: Reviewer comments [file LSA-2024-02875_review_history.pdf]

Life Science Alliance

Correction: Apolipoprotein E intersects with amyloid-β within neurons

Sabine Konings, Emma Nyberg, Isak Martinsson, Laura Torres-Garcia, Oxana Klementieva, Claudia Guimas Almeida, and Gunnar Gouras
DOI: https://doi.org/10.26508/lsa.202402875

Corresponding author(s): Gunnar Gouras, Lund University

Review Timeline:

Submission Date:	2024-06-06
Accepted:	2024-06-10

Scientific Editor: Eric Sawey, PhD

Transaction Report:

(Note: With the exception of the correction of typographical or spelling errors that could be a source of ambiguity, letters and reports are not edited. The original formatting of letters and referee reports may not be reflected in this compilation.)

June 10, 2024

RE: Life Science Alliance Manuscript #LSA-2024-02875

Prof. Gunnar K. Gouras
Lund University
Experimental Medical Science
Experimental Dementia Research Unit
Sölvegatan 19, BMC B11
Lund 22184
Sweden

Dear Dr. Gouras,

Thank you for submitting your Correction entitled "Correction: Apolipoprotein E intersects with amyloid-β within neurons".

IMPORTANT: If you will be unreachable at any time, please provide us with the email address of an alternate author. Failure to respond to routine queries may lead to unavoidable delays in publication.

You can contact the journal office with any questions, contact@life-science-alliance.org

Sincerely,

Eric Sawey, PhD
Executive Editor
Life Science Alliance
http://www.lsajournal.org

June 10, 2024

RE: Life Science Alliance Manuscript #LSA-2024-02875

Prof. Gunnar K. Gouras
Lund University
Experimental Medical Science
Experimental Dementia Research Unit
Sölvegatan 19, BMC B11
Lund 22184
Sweden

Dear Dr. Gouras,

Thank you for submitting your Correction entitled "Correction: Apolipoprotein E intersects with amyloid-β within neurons".

IMPORTANT: If you will be unreachable at any time, please provide us with the email address of an alternate author. Failure to respond to routine queries may lead to unavoidable delays in publication.

You can contact the journal office with any questions, contact@life-science-alliance.org

Sincerely,

Eric Sawey, PhD
Executive Editor
Life Science Alliance
http://www.lsajournal.org
